# Rhabdomyolysis following Acute Extended-Release Quetiapine Poisoning: A Case Report

**DOI:** 10.1155/2012/347421

**Published:** 2012-12-13

**Authors:** Antonios Liolios, Othman Sentissi

**Affiliations:** Department of Mental Health and Psychiatry, University Hospitals of Geneva, 35 Rue des Bains, 1205 Geneva, Switzerland

## Abstract

*Background*. During the past few years, there have been a number of case reports concerning rhabdomyolysis following quetiapine poisoning; however, there has been none concerning the medication in its extended-release form. *Methods*. We present the case report of a 48-year-old man presenting a major depressive disorder and borderline personality disorder, who after voluntary intoxication with 12000 mg of quetiapine extended-release developed signs of acute rhabdomyolysis. *Results*. The rhabdomyolysis was confirmed by the laboratory and the clinical findings, with elevated levels of creatinine, creatine phosphokinase, and CRP. *Discussion*. We would like to pinpoint the importance of this complication and our concern of prescribing it for psychiatric patients with chronic somatic comorbidities.

## 1. Background

During the past few years, there have been several reports concerning the clinical features of quetiapine poisoning (such as hypotension, tachycardia, QT prolongation, seizures, arrhythmia, neuroleptic malignant syndrome, and death) [1–3] with few of them concerning episodes of severe rhabdomyolysis [4–7]. Rhabdomyolysis has been shown to be a well-known cause of acute renal failure; amongst the most frequent causes are trauma, alcohol and drug abuse, prolonged immobilization, as well as exertion during exercise, although diverse causes have been mentioned [8]. However, to our knowledge this is the first rhabdomyolysis case report described for an acute quetiapine poisoning episode in its extended-release form, concerning a patient who has not been suffering from a psychotic condition (as described in most case reports).

## 2. Method and Results

We report the case of a 48-year-old man of Swiss-French origin, diagnosed with major depressive disorder and borderline personality disorder, with a history of multiple suicide attempts by medication overdose. In general good health, the patient had developed another episode of rhabdomyolysis in 2009 accompanied by acute renal failure, possibly by dehydration (a voluntary ingestion of quetiapine, escitalopram, and other psychotropic medications were suspected but not confirmed). The treatment of this patient, who has been in our outpatient clinic for more than 8 years, consists of benzodiazepinic sedatives (lorazepam), antidepressants (mirtazapine), hypnotics (flurazepam), and quetiapine in the extended-release form (XR), his prescribed dosage being 400 mg once daily. This treatment had been well tolerated for at least one year, without any adverse reactions.

According to the patient, he took about 12000 mg of quetiapine (a package of 30 pills of 400 mg) in a suicide attempt, without taking any other of his medications, falling unconscious shortly on his couch. He woke up nearly 24 hours later. On his arrival at the emergency department where he presented by himself, he was barely able to walk, presenting with a large bruise on his buttocks and minor scratches on his lower extremities. He presented short-term memory loss, not having any clear recollection of the incident. He denied any incontinence (urinary or fecal) or biting his tongue; furthermore, the patient has no history of seizures. No abuse of alcohol or other drugs was reported. On clinical examination, the vital signs were blood pressure at 130/90 mm Hg, pulse rate at 111 bpm, and temperature at 36.8°C. No clinical signs of infection were present; leucocytes were at 9.7 G/L (normal intervals at 4–10 G/L). The ECG was normal, with a normal QT interval (at 0.41 mm/s). Laboratory data was creatine phosphokinase (CPK) at 4005 U/L, (normal values < 145 U/L) creatinine 110 U/L (normal values at 53–97), and C-Reactive Protein at 133.8 mg/L (normal values at 0–10). During a psychiatric assessment in the emergency department, he acknowledged the suicidal intent of his intoxication and he denied the ingestion of any other of his treatments. He was discharged by our colleagues and referred to our day clinic.

Forty-eight hours after the incident, a further lab test confirmed the rhabdomyolysis, with values of creatine phosphokinase 3780 U/L, lactate dehydrogenase 483 U/L, gamma-GT 138 U/L (normal intervals < 38), creatinine 99 micromol/L, CRP 87,1 mg/L, and leucocytes 10.6 G/L. Blood quetiapine levels were normal. Normal blood levels of creatine phosphokinase, creatinine, and CRP were confirmed a week later through a further blood test. 

## 3. Discussion

The Swiss Pharmacological guide [9] mentions a risk of diffuse muscular pain in therapeutic dosage of quetiapine; however, this was not the case of our patient, who tolerated well the medication. It is also noteworthy that 2 months before the incident described above, the GP of the patient prescribed a statin (a known cause of rhabdomyolysis) [7] at 20 mg/day for his high cholesterol levels; however, this treatment was stopped with the doctor's consent some days later because of muscular pain in the lower extremities. The dose of quetiapine ingested by the patient had a strong sedative and hypnotic effect, which provoked immobility— a well-known cause of rhabdomyolysis; however, the absence of muscle wasting, as well as absence of pain in the extremities makes a direct toxic effect of quetiapine poisoning more probable in this case. We can also exclude the presence of a neuroleptic malignant syndrome (absence of neurological signs and symptoms, and hyperthermia, autonomic dysfunction).

To our knowledge, this is the first case of rhabdomyolysis following acute quetiapine poisoning by the extended-release form of quetiapine, for this patient suffering from a nonschizophrenic condition. Considering the frequent use of quetiapine for bipolar spectrum disorders [10] and borderline personality disorders [11], we would like to pinpoint the importance of this complication, especially for psychiatric patients with somatic comorbidities (e.g., chronic renal failure); careful assessment of renal function should be undertaken. 
